# First Molecular Detection of Pathogens *Leptospira* in Common Rodent Captured in North Algeria Urban Areas

**DOI:** 10.3390/tropicalmed7110335

**Published:** 2022-10-29

**Authors:** Lila Lekhal, Elena Harran, Anaïs Aragon, Karine Groud, Marine Le Guyader, Rachid Kaidi, Djamel Khelef, Zouheira Djelouadji

**Affiliations:** 1 Laboratoroire des Biotechnologies Liées à la Reproduction Animale, Institut des Sciences Vétérinaires, Univerité Saad Dahlab Blida1, P.B. 270, Route de Soumaa, Blida 09000, Algeria; 2 Départment des Sciences Vétérinaires, Institut des Sciences Vétérinaires, Université Saad Dahlab Blid1, Blida 09000, Algeria; 3 USC1233-INRAe Rongeurs Sauvages, Risque Sanitaire et Gestion des Populations, VetAgro Sup, Campus Vétérinaire de Lyon, 69280 Marcy l’Etoile, France; 4 Faculty of Arts and Sciences, Holy Spirit University of Kaslik (USEK), Jounieh P.O. Box 446, Lebanon; 5 Ecole Nationale Supérieure Vétérinaire, Oued Smar, Alger 16000, Algeria; 6 Laboratoire de Santé et Production Animale, Ecole Nationale Supérieure Vétérinaire, Oued Smar, Alger 16000, Algeria

**Keywords:** leptospirosis, Algeria, Blida, rodents, *L. interrogans*, *L. borpetersenii*, *Rattus Norvegicus*, *Rattus Rattus*, *Mus Musculus*

## Abstract

Leptospirosis is an anthropozoonosis disease of worldwide distribution caused by mobile spirochetes of the genus *Leptospira* and rodents, mainly rats, are described as its primary reservoir. In Algeria, there is limited data about the prevalence of *Leptospira* spp. in humans and animals, as well as *Leptospira* carriage in wild rodents. The study aimed to highlight the importance of rodents as a reservoir of *Leptospira* bacterium in Blida city in Algeria by detecting and identifying circulating *Leptospira* species in the rodent population. A total of 101 rodents, 95 *Rattus Norvegicus*, 5 *Rattus Rattus*, and 1 *Mus Musculus* were captured and tested for pathogenic *Leptospira* spp. byreal-time PCR targeting the *Leptospira* 16S rRNA (rrs) gene, revealing a total prevalence of 40.6%, 95% IC [30.9–50.8%]. Positive samples were subjected to species-specific real-time PCR assays targeting *L. interrogans*, *L. noguchii*, *L. borgpetersenii*, and *L. kirschneri* for species identification. However, positive samples for which *Leptospira*-species could not be determined were subjected to conventional PCR targeting the partial 16S rRNA (rrs) gene, and amplified DNA was subjected to sequencing. *Leptospira* spp. was detected in 36 kidney, 16 urine, and three lung specimens. *L. interrogans* was identified in 39 rodents and *L. borpetersenii* in one rodent; however, one rodent with renal carriage could not be typed due to poor DNA quality. This study provides the first description of pathogenic *Leptospira* spp. in wild rodents in Algeria. These findings suggest a high potential risk of leptospirosis transmission from rodents to humans and animals in Algeria and therefore imply the adoption of prophylactic measures. In addition, further studies, including different animals and rodent species, should be conducted to clarify the epidemiology of this disease in Algeria.

## 1. Introduction

Leptospirosis is an emerging rodent-borne disease [1] most commonly found in tropical and subtropical regions [2]. It affects more than one million people annually, with 60,000 deaths recorded [3]. Infection in humans and animals is caused by pathogenic *Leptospira* spp. [4]. Leptospires are divided into more than 300 serovars, grouping 25 serogroups [5,6] and 69 genomic species [7], including four species that have been recently isolated from fresh water in Algeria [8]. Leptospires are maintained in several wild and domestic animal hosts [2] through the renal carriage and are excreted in the urine for several months [9]. Infection in humans and animals mainly occurs through contact with tissues of infected mammalian hosts, mainly rodents, or indirect exposure to soil or water contaminated by leptospires [10].Rodents are considered the most important reservoir of pathogenic *Leptospira* spp. worldwide due to their close contact with humans and domestic animals [11].

*L. interrogans*, *L. borgpetersenii*, and *L. kirschneri* are the most abundant species circulating in humans and animals worldwide [12], with *L. interrogans* being the most described in rodents [13] and recognized as the most common species infecting humans [14]. Consequently, long-term active surveillance and investigations of the *Leptospira*-carriage status of rodents can contribute to understanding animal-to-human transmission, investigating outbreaks, and identifying source populations of leptospirosis [15].

In humans, clinical manifestations of leptospirosis range from mild febrile illness (in 90% of cases) [16] to life-threatening renal failure, pulmonary hemorrhage, and/or cardiac complication [17]. In dogs, clinical manifestations vary from asymptomatic to hepatic and renal failure, with hemorrhagic and pulmonary disorders frequently occurring [18]. In livestock, the disease causes reproductive failures and economic losses [19] and recurrent uveitisis observed in horses [20]. 

Data on circulating leptospires varies between regions [13]. Few data, mostly serological, describe the circulating strains of leptospires in Algeria. Two unique studies performed the Microscopic Agglutination Test (MAT) on humans. The first was conducted between 2006 and 2007 in the region of Tizi Ouzou and revealed human infection by serogroup Icterohemorrhagiae in more than 60% of the cases [21]. The second was conducted between 1 January 2005 and 31 December 2008 in the infectious diseases department of the University Hospital of Tizi-Ouzou. It brought together 173 hospitalized patients and revealed the predominance of serogroups Icterohemorragiae and Canicola in infected patients [22].

Regarding domestic animals, a recent study relative to the urinary carriage of leptospires in stray dogs and cats in the Algiers region was carried out in 2018 [23] and demonstrated the occurrence of *L. interrogans* in 5 of 107 dogs’ urine by PCR. In the same region, a second study in 2017 investigated the causes of abortion in dairy cattle through serological screening of 360 cattle from 54 farms and reported *L. interrogans* serovarHardjo in 14 cows [24]. Finally, a recent study carried out on cattle between 2015 and 2019 in the state of Setif in northeastern Algeria performed an Indirect Enzyme-Linked Immunosorbent Assay on 406 cattle from 48 herds and reported *L.interrogans* serovar Hardjo in 31.25% of the herds and 5.42% of the cattle [25].

To the best of our knowledge, no study describing *Leptospira-*carriage in wild rodents has been carried out in Algeria until today. Nevertheless, a unique study was conducted in some farms of the Algiers region in which twenty *Rattus Norvegicus* were trapped and investigated for leptospirosis by serology (MAT) and culture of kidney and urine. Results of the latter study showed that only 2 of the 20 rats included in the study showed (low) antibody titers of 1:40 and 1:160 against serovars Gripotyphosa and Australis, respectively, by MAT. Furthermore, no evidence of the bacterium was revealed by culture [26]. Consequently, knowledge of the circulation of *Leptospira* strains in wild rodents is lacking in Algeria. Therefore, information regarding leptospirosis and *Leptospira* circulating serogroups in Algeria are required to implement appropriate prophylaxis measures, if needed. The objectives of the present study were to assess rodent *Leptospira* carriage rates in Blida, a central northern city of Algeria, and identify circulating *Leptospira* species.

## 2. Materials and Methods

### 2.1. Regulatory and Ethical Aspects

Rodents were captured in collaboration with the Service of Hygiene of Blida under the authorization number BHC/340/2018. Rodents were anesthetized and euthanized in accordance with the ethical standard of the Laboratory of Animal Reproduction Biotechnologies (LBRA), Institute of veterinary science, university Blida1, under the authorization number (98/LBRA/Univ Blida/20). The species included in this study (*RattusNorvegicus*, *Rattus rattus*, and *Mus musculus*) are classified as species of the least concern on the IUCN list [27,28,29], which have the lowest level of protection in this classification.

### 2.2. Rodent’s Capture 

**Figure 1:**
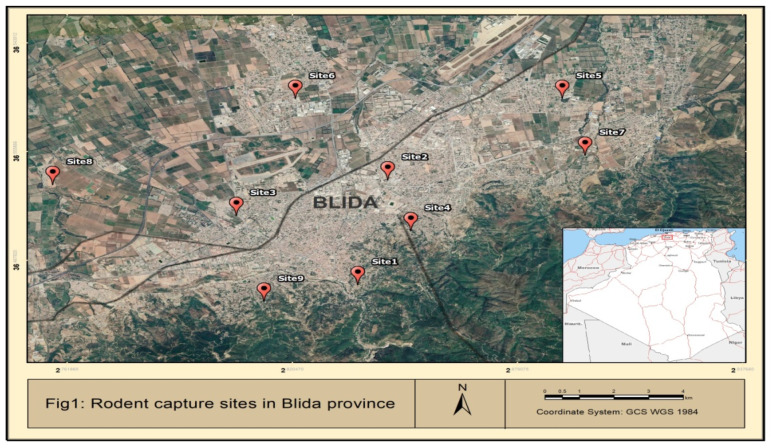
Geographic localization of the sites of capture.

Rodent’s capture campaign took place in the region of Blida, Algeria (Figure 1) between 11 November 2018 and 9 June 2020. A total of nine distinct sites were targeted, including six urban areas (Site 1: 36°27′45″ N 2°50′15″ E, Site 2: 36°29′57″ N 2°50′43″ E, Site 3: 36°28′02.6″ N 2°49′16.3″ E), Site 4: 36°28′52.8″ N 2°51′04.5″ E, Site 5: 36°31′39.4″ N 2°53′26.4″ E, Site 6: 36°29′12″ N 2°48′21″ E, two rural areas (Site 7: 36°30′28″ N 2°53′48″ E, Site 8: 36°29′51″ N 2°45′29″ E) and one peri-urban environment (Site 9: 36°27′24″ N 2°48′47″ E) (Figure 1). The sites were chosen depending on different criteria such as site accessibility and safety, availability of personnel, help accorded by the owners, and the existence of rodents. The trapping was conducted in one session and for one month solely for each site, except for sites 1 and 9 (three sessions during twelve months for site 1 and two sessions during six months for site 2).Most of the rodents were captured during the rainy season between October and April. Rodents were captured using spring cages of suitable size for rats (approximately 30 cm× 15 cm× 15 cm). Each site had five to ten traps along the walls at intervals of two meters. The traps were collected every morning, and the captured rodents were promptly transferred to the inspection site and sacrificed. All cages were cleaned prior to being reinstalled. Furthermore, rodent species were determined according to demographic characteristics such as body measurements (head and body length, tail and skull length, foot and ear length) and fur color [30]. The sex and age of each animal were also recorded [31].

### 2.3. Collecting Samples

Following their euthanasia and the disinfection of the abdomen with alcohol, rodents were autopsied. Kidneys and lungs were collected and stored at −20 °C. When possible, urine samples were collected directly from the bladder of the animal using a 1mL sterile syringe. The samples were then sent to the USC1233 Unit at VetAgroSup, veterinary campus of Lyon, France, in cold conditions in less than 24 h.

### 2.4. DNA Extraction

DNA was extracted from samples using the QIAamp DNA Mini Kit from Qiagen (Qiagen, Courtaboeuf, France) following the manufacturer’s recommendations.

Initially, pretreatment of the samples was carried out. Kidneys and lungs were rinsed with sterile PBS, and a portion of 20 to 25 mg from each tissue (targeting the cortico-medular area for kidneys) was used for DNA extraction. The tissue portions were crushed using a 5 mL syringe and added to an Eppendorf tube containing 180 µL of T1 lysis buffer and 20 µL of Proteinase K. After being vigorously vortexed, samples were incubated 1 to 3 h at 56 °C in a dry bath until the complete and homogeneous dissolution of the tissues. Samples were again vortexed, and then 200 μL of B3 lysis buffer was added. The negative control of extraction or NCS (Negative Control Sample) was prepared in an Eppendorf tube containing all added reagents without sample tissue. For urine, 1 mL was centrifuged for 10 min at 4000 rpm in a sterile Eppendorf tube. The supernatant was discarded, then the pellet was washed with 1 mL of PBS. After a second centrifugation for 10 min at 4000 rpm, the pellet was collected with 200 µL of lysis buffer B3 and added to an Eppendorf tube containing 25 µL of proteinase K. The NCS was prepared in an Eppendorf tube containing all added reagents with 200 μL of PBS.

After carrying out the appropriate pretreatment, all samples were vortexed and incubated for 10 to 15 min at 70 °C in a dry bath. Then, 200 µL of 100% ethanol was added before vortexing again. Samples were transferred to the extraction columns on a silica membrane and centrifuged for 1 min at 11,000 rpm. The columns were then washed twice by centrifuging for 1 min at 11,000 rpm, first with 500 μL of BW buffer and then with 600 μL of B5 buffer. The collection tubes were changed following each wash to avoid contamination. The columns were dried by centrifuging in vacuo again for 1 min at 11,000 rpm, then sterile Eppendorf tubes were added under the columns. Finally, 50 µL of BE buffer preheated to 70 °C was added to columns to allow DNA elution. After 1 min of impregnation at room temperature, columns were centrifuged for 1 min at 11,000 rpm, and the eluted DNAs were collected.

### 2.5. Detection and Identification of Pathogenic Leptospires by Real-Time PCR Assays

The detection of pathogenic leptospires was performed by real-time PCR targeting a partial region of the *Leptospira* 16S rRNA (rrs) gene using primers and probes as described by Waggoner et al. [32] (Table 1).

*Leptospira*-positive samples were subsequently tested with the individual species-specific assays (for a total of four probe/primers sets) targeting respectively *L. interrogans*, *L. noguchii*, *L. borgpetersenii* and *L. kirschneri* (Table 1) [33]. The amplification reactions were optimized individually for all probes and associated primers using the AgPath-ID™ One-Step RT-PCR Reagents (Life technologies, Courtaboeuf- Les Ulis, France), according to the manufacturer’s instructions. The Mx3000P real-time PCR detection system (Agilent Technology, Courtaboeuf- Les Ulis, France) was used for all assays. Each reaction was conducted in a total volume of 25 µL consisting of 2X RT-PCR Buffer, 300 nM of each primer, 400 nM of TaqMan probe, and 4 µL of DNA template solution. Amplification conditions were: 95 °C for 10 min, followed by 40 cycles of 15 s at 95 °C and 1min at the optimized annealing temperature for each probe. Positive and negative controls were included in each PCR run. The positive control consisted of the mixture with *Leptospira* DNA as a template: *L. interrogans* Icterohaemorrhagiae 19 ENVN, *L. noguchii* Panama panama, *L. borgpetersenii* Sejroe Sejroe M84, and *L. kirschneri* GrippotyphosaGrippotyphosa Moskva-V, for *L. interrogans*, *L. noguchii*, *L. borgpetersenii*, and *L. kirschneri* respectively, and the negative control consisted of 5 µL sterile water added to a well containing the same mixture. Data analyses were performed by the detection system of the real-time PCR equipment, according to the manufacturer’s instructions.

### 2.6. Conventional PCR and Sequencing

*Leptospira*-positive samples that could not be typed by the individual species-specific real-time PCR were prone to conventional PCR, and amplified DNA was subjected to sequencing.

A conventional PCR targeting the partial 16S gene, encoding the subunit of the 16S ribosomal RNA, was performed. Amplification and sequencing of this partial region (330 bp) allow the detection and identification of the genomic species of pathogenic leptospires [34]. PCRs were carried out using the HotStarTaq DNA Polymerase from Qiagen (Qiagen Courtaboeuf-Les Ulisville, France). Each reaction was conducted in a total volume of 25 µL consisting of H2O, 10x PCR Buffer, MgCl2, dNTP mix, Primer F at 10 μM (sequence 5’-3’: GGCGGCGCGTCTTAAACATG), Primer R at 10 μM (sequence 5’-3’: TTCCCCCCATTGAGCAAGATT), GoTaq G2 Hot Start Polymerase and 5 µL of DNA template solution. Positive and negative controls were included in the PCR run as described above. The PCR program consisted of an initial denaturation step at 95 °C for 15 min, followed by 40 cycles each, including a second denaturation step at 95 °C for 30 s, a hybridization step at 57 °C for 30 s and an elongation step at 72 °C for 1 min, and a final elongation step at 72 °C for 10 min.

The amplified DNAs were revealed by electrophoresis on 1.5% agarose gel prepared from TBE 10X buffer. Reading was carried out using a UV reader following 20 min DNA migration at 135 volts.

The 16S conventional PCR DNA positive samples were prone to sequencing by Genoscreen, Lille, France platform. The sequences obtained were adjusted using ChromasProsoftware. Finally, sequences were compared to the GenBank database on the NCBI (National Center for Biotechnology Information) website using the BLAST (Basic Local Alignment Search Tool) [35] and to an internal database, and genomic species were identified by homology.

## 3. Results

### 3.1. Capture of Rodents

One hundred and one (101) rodents were captured, including 95 *Rattus Norvegicus*, 5 *Rattus Rattus*, and 1 *Mus Musculus*. Among them, fifty-two (52/101, 51.5%) were males, and forty-nine (49/101, 48.5%) were females. We identified eighty-nine (89/101, 88%) adults, four (4/101, 4%) sub adults and eight (8/101, 8%) young rodents. The majority of rodents were captured in the urban area (62/101, 61.4%), whereas twenty-seven (27/101, 26.7%) were captured in peri-urban areas, and twelve (12/101, 11.9%) in the rural area (Table 2).

### 3.2. Detection of the Leptospira DNA by Real-Time PCR

Forty (40/95, 42.1%, 95% IC [32–52.7%]) *Rattus Norvegicus* and one (1/5, 20%, 95% IC [0.5–71.6%]) *Rattus Rattus* revealed positivity in at least one of the sampled specimen by real-time PCR (Table 3).

In total, 52 specimens were positive, counting thirty-six (36/101, 35.64%) kidneys, thirteen (13/24, 54.2%) urines, and three (3/101, 3%) lungs (Table 4) (Table S1).

According to rodents’ gender, twenty-four (24/52, 46.2%) males and seventeen (17/49, 34.7%) females were positive. Regarding the age group, thirty-nine (39/89, 44.1%) adults, one (1/4, 25%) sub adult, and one (1/8, 12.5%) young rodents were positive. Moreover, twenty-eight (28/62, 45.2%) captured rodents in urban areas, ten (10/27, 37%) captured in peri-urban areas, and three (3/12, 25%) in rural areas were positive. 

### 3.3. Leptospira Species Identification

*Leptospira* species identification was performed on all positive DNA samples by individual species-specific PCR assays targeting *L. interrogans*, *L. noguchii*, *L. borgpetersenii*, and *L. kirschneri*. Of the 52 positive samples, 42 could be identified as *L. interrogans*, including thirty (30/36) kidney, eleven (11/13) urine, and one (1/3) lung samples. However, 8 of the 52 positive samples, including five (5/36) kidneys, one (1/13) urine and two (2/3) lungs, could not be typed using species-specific PCR (Table 4). Of these eight samples, seven (7/8) were identified as *L. interrogans* and one (1/8) as *L. borpetersenii* by sequencing. However, two positive DNA samples, one (1/13) urine and one (1/36) kidney, could not be typed due to poor DNA quality (Table S1, Figure S1). In total, *L. interrogans* and *L.* *borpetersenii* were identified in 49 and one positive DNA sample, respectively (Table 5).

## 4. Discussion

To the best of our knowledge, this is the first study to describe *Leptospira*-carriage in wild rodents in Algeria, highlighting the need to develop effective prophylaxis measures in the nation to ensure human and animal protection. 

Among the one hundred and one (101) captured rodents, forty-one (41/101) were carrying leptospires in at least one of the sampled specimens, and the total prevalence of infected rodents was 40.6%, 95% [30.9–50.8%]. The prevalence reported in this study is relatively high when compared with the prevalence reported in wild rodents in Africans and Mediterranean countries; in Niger (1.3%, 8/578) [36], in Egypt, (19%, 19/100) [37]; (24%, 65/270) [38], in Kenya (18.3%, 41/224) [39], in Spain (10.52%, 2/19) [40] and in France (10.43%, 12/115) [41]. Nevertheless, our prevalence is slightly higher than 35.2% (81/230) reported in rodents in Italy [42].

Three of forty-one (3/41) positive rodents belonging to *Rattus Norvegicus* presented pulmonary carriage. The pulmonary localization of *Leptospira* spp. in wild rodents has only been described once in the literature following the serovar *Icterohaemorrhagiae* on the ciliated epithelium of the bronchi of 3 *Rattus Norvegicus* trapped in the Lyonnaise region, in France [43]. Experimental infection of R. Norvegicus with L. interrogans demonstrated the persistence of leptospires for several months in kidneys and its elimination from other tissue, including lungs, within a maximum of 9 days [44].

Among rodents for which urine specimen was collected, four revealed *Leptospira* in urine solely. This finding was unexpected, given that urine excretion results from the renal carriage. Nevertheless, leptospires are recognized to colonize a defined number of niches in kidney specimens proportional to the infecting dose [45]. However, only a limited portion (20-25mg) of (kidney) specimen was used in the DNA extraction step. This might have resulted in a negative PCR due to the localization of leptospires on a specific portion of the kidney (not collected), making the actual prevalence underestimated.

According to rodent species, forty (40/95) *Rattus Norvegicus* and one (1/5) *Rattus Rattus* reported a prevalence of 42.1%, 95% IC [32–52.7%] and 20%, 95% IC [0.5–71.6%], respectively. Consequently, *Rattus Norvegicus* revealed a higher prevalence than *Rattus Rattus*. The latter result is consistent with studies carried out in Benin [46] (*Rattus Norvegicus*; 27.3%, *Rattus Rattus*; 13.3%), Singapore (*Rattus Norvegicus*; 46.8%, *Rattus Rattus*; 10.4%) [47] and Mahé Island, Seychelles (*Rattus Norvegicus*; 54.0%, *Rattus Rattus*; 4.4%) [48]. Furthermore, our finding makes sense given that *R. rattus* favors dry habitats, whereas *R. norvegicus* favors wet habitats [49], with garbage dumps and sewers, where leptospires are found in the majority, explaining the strong association between *R. norvegicus* and the high prevalence of *Leptospira* spp. [47].

Males were more exposed to *Leptospira* infection (46.2%) than females (34.7%); our result is in accordance with studies conducted on rodents in Kenya and Salvador, where 17.9% [39] and 97% of *Leptospira*-positive rats were males [50]. In contrast, a study in Austria [51] reported that female rats were more infected (37.5%) than males (18%). Furthermore, the proportion of positive individuals increased with the age of rodents, with *Leptospira* being identified in 44.1% of adults, 25% of sub adults, and 12.5% of juveniles. Our result is compatible with the one reported in studies conducted on rodents in Vancouver and Salvador [52], in Brazil [53], and in Salvador, Bahia [50]. Physiological, immunological, and behavioral characteristics associated with age are reported to play an important role in the transmission of infections [54]. Effectively, males and adults of *R. norvegicus* have a social behavior of huddling and fighting, along with an active search for food [55]. Aggressive behavior, such as fighting and biting, is known to facilitate the transmission of *Leptospira* infection [56]. The proportion of positive individuals also increased with urbanization (45.2% in urban areas, 37% in peri-urban areas, and 25% in rural areas). Rats living in rural habitats commonly grow at a lower rate, whereas the abundance of nutritional sources with less competition for it in urban areas and the abundance of possible shelters afforded by urban landscapes promote the proliferation of rats [57,58]. Consequently, the high density of rats in urban areas associated with stagnant water could ease the spread of leptospires among rodents in urban areas [13].

Finally, the genomic *leptospira* species was investigated by the individual species-specific real-time PCR assays targeting *L. interrogans*, *L. noguchii*, *L. borgpetersenii*, and *L. kirschneri* or by sequencing the amplified DNA of the 16S gene. *L. interrogans* was identified in 49 of the 52 positive sample specimens corresponding to 39 individuals. However, *L. borpetersenii* was identified in one kidney sample corresponding to one individual. *L. interrogans* is described as the most common genomic species in wild rats (*Rattus* spp.) worldwide [13] and the most frequent species infecting humans [14]. In Algeria, *L. interrogans* was implicated in many cases of human leptospirosis between 2005 and 2008 [21,22] and was identified in 5 dogs in 2017 in northern Algeria [23]. *L. borpetersenii* was found in a single individual in our survey; this species was also associatedwith rats species; *Rattus Norvegicus* in South Africa [59] and Madagascar [14] and *Rattus Rattus* in Sardinia [49] and Japan [60]. The low rate of carriage of *L. borpetersenii* in rodents observed in the present study can be explained by the fact that *L. borpetersenii* colonizesfewer rat kidneys [61] when compared to *L. interrogans* known to persist for several months in kidneys [44]. *L. interrogans* transmission commonly occurs via contaminated surface water, whereas epidemiological data support a host-to-host transmission cycle for *L. borgpetersenii* [62].

## 5. Conclusions

The present study revealed the presence of *Leptospira* spp. in wild rodents in Algeria with the identification of *L. interrogans* and *L. borgpetersenii* infectious species. The study highlighted the importance of rodents as a potential reservoir of *Leptospira* in northern Algeria, constituting a high risk of transmission to humans and animals, therefore adapting effective prophylactic measures. However, since the rodents included in this study derived from the region of Blida (Algeria), the identified *Leptospira* strains may not be typical of those present in other parts of Algeria. Thus, further studies should be carried out on rodents and other animal species in the rest of the country in order to clarify the circulating *Leptospira* strains.

## Figures and Tables

**Table 1:** Primers and probes used for real-time PCR.

Prime/Probe	Sequence5′-3′	Targeted Gene
Forward Reverse Pathogen probe	CGGGAGGCAGCAGTTAAGAA CGTATGGTGCAAGCGTTGTT GCAATGTGATGATGGTACCTGCC	*Leptospira* 16S rRNA (rrs) gene [32]
PFLint2 PRLint2 TaqLint2	CTT GAG CCT GCG CGT TAY C CCG ATA ATT CCA GCG AAG ATC TET-CTC ATT TGG TTA GGA GAA CAG ATC A-BHQ1	secY gene of *L. interrogans* [33]
F_bpn R_bpn1 TqM_bpn	GAT TCG GGT TAC AAT TAG ACC TTG ATC TAA CCG GAC CAT AGT Cy5.5 (Quasar 705)-TAC TAA GGA TGG TTT GGA CGC TGC-BHQ2	ompL1 gene of *L. borgpetersenii* [33]
F_nery R_nery TqM_nery	CTG GCT TAA TCA ATG CTT CTG CTC TTT CGG TGA TCT GTT CC Texas Red-CAG TTC CAG TTG TAA TAG ATA AGA TTC-BHQ2	secY gene of *L. kirschneri* [33]
FLnog2 RLnog2 TaqLnog	TCA GGG TGT AAG AAA GGT TC CAA AAT TAA AGA AGA AGC AAA GAT FAM-CGA TTG GCT TTT TGC TTG AAC CATC-BHQ1	secY gene of *L. noguchii* [33]

**Table 2:** Distribution of number of positive rodents according to their features.

		Total Number	Positive Number
Gender	Female	49	17
	Male	52	24
Age	Adult	89	39
	Subadult	4	1
	Young	8	1
Habitat	Urban	62	28
	Peri-urban	27	10
	Rural	12	3

**Table 3:** Prevalence of *Leptospira* in the rodent species.

		Total Number	Positive Number	Prevalence
Rodent species	*Rattus Norvegicus*	95	40	42.1%, 95% IC [32–52.7%]
	*Rattus Rattus*	5	1	20%, 95% IC [0.5–71.6%]
	*Mus Musculus*	1	0	0%, 95% IC [0–97.5%]
	Total	101	41	40.6%, 95% IC [30.9–50.8%]

**Table 4:** Distribution of positive rodent specimens according to *Leptospira* species identification method.

Rodent Specimen (n)	Total Positive	Species-Specific PCR Identification	Sequencing *	Unknown
Kidney (101)	36	30	5	1
Lung (101)	3	1	2	0
Urine (24)	13	11	1	1
Total(226)	52	42	8	2

(*) Sequencing made following conventional PCR confirmation.

**Table 5:** The total number of *Leptospira* species identified using species-specific PCR identification or DNA sequencing.

	Number of *Leptospira* Species Identified		
Rodent Positive Specimen (n)	*L. interrogans*	*L.borgpetersenii*	Unknown
Kidney (36)	34	1	1
Lung (3)	3	0	0
Urine (13)	12	0	1
Total (52)	49	1	2

## Data Availability

The data used to support this study are available from the corresponding author upon request.

## Supplementary Materials

The following supporting information can be downloaded at: https://www.mdpi.com/article/10.3390/tropicalmed7110335/s1. Figure S1: Flow diagram summarizing all PCRs reactions, and Table S1: Detailed information of all positive samples for *Leptospira* species identification.
