# Supplementary material for: First Molecular Detection of Pathogens Leptospira in Common Rodent Captured in North Algeria Urban Areas
Source: Trop Med Infect Dis. 2022 Oct 29;7(11):335. doi: 10.3390/tropicalmed7110335 (PMC9693108; doi:10.3390/tropicalmed7110335)
Supplement: Supplementary file 1 [file tropicalmed-07-00335-s001.zip › Table S1.pdf]

Table S1. Detailed information of all positive samples for *Leptospira* species identification.

	Rodent's species[a]	Sexe	Age	Site[b]	Kedney *Leptospira* species identification	Lung *Leptospira* species identification	Urine *Leptospira* species identification
**Rat 1**	*RN*	Male	Adult	1 urban	***L. interrogans****	-	-
**Rat 7**	*RN*	Male	Adult	1 urban	*L. interrogans*	*L. interrogans*	unknown
**Rat 8**	*RN*	Female	Adult	1 urban	*L. interrogans*	-	-
**Rat 9**	*RN*	Male	Adult	1 urban	*L. interrogans*	-	-
**Rat 10**	*RN*	Male	Young	1 urban	***L. interrogans****	-	-
**Rat 14**	*RN*	Female	Adult	1 urban	*L. interrogans*	-	-
**Rat16**	*RN*	Female	Adult	9 periurban	*L. interrogans*	-	*L. interrogans*
**Rat 19**	*RN*	Female	Adult	9 periurban	-	-	*L. interrogans*
**Rat 20**	*RN*	Male	Adult	9 periurban	*L. interrogans*	-	-
**Rat 25**	*RN*	Female	Adult	9 periurban	*L. interrogans*	-	-
**Rat26**	*RN*	Male	Adult	9 periurban	*L. interrogans*	-	-
**Rat29**	*RN*	Male	Adult	1 urban	-	-	***L. interrogans****
**Rat 30**	*RN*	Female	Adult	1 urban	*L. interrogans*	-	-
**Rat 34**	*RN*	Female	Adult	1 urban	*L. interrogans*	-	-
**Rat 41**	*RN*	Female	Adult	9 periurban	*L. interrogans*	-	-
**Rat 46**	*RR*	Male	Adult	9 periurban	***L. interrogans****	-	-
**Rat 48**	*RN*	Male	Adult	9 periurban	***L.borgpetersenii****	-	-
**Rat 51**	*RN*	Female	Adult	9 periurban	*L. interrogans*	-	-
**Rat 52**	*RN*	Male	Adult	9 periurban	***L. interrogans****	-	-
**Rat 55**	*RN*	Male	Adult	2 urban	*L. interrogans*	-	*L. interrogans*
**Rat 57**	*RN*	Female	Adult	7 rural	*L. interrogans*	-	-
**Rat 58**	*RN*	Male	Adult	7 rural	*L. interrogans*	-	*L. interrogans*
**Rat 59**	*RN*	Male	Adult	7 rural	*L. interrogans*	-	*L. interrogans*
**Rat 62**	*RN*	Male	Adult	2 urban	unknown	-	-
**Rat 66**	*RN*	Female	Adult	6 urban	*L. interrogans*	-	-
**Rat 70**	*RN*	Female	Adult	2 urban	*L. interrogans*	-	*L. interrogans*
**Rat 73**	*RN*	Male	Adult	1 urban	*L. interrogans*	-	-
**Rat 74**	*RN*	Female	Adult	6 urban	*L. interrogans*	-	-
**Rat 75**	*RN*	Male	Adult	6 urban	*L. interrogans*	***L. interrogans****	-
**Rat 76**	*RN*	Male	Adult	2 urban	*L. interrogans*	-	-
**Rat 77**	*RN*	Male	Adult	2 urban	-	***L. interrogans****	-
**Rat 80**	*RN*	Female	Sub-adult	1 urban	*L. interrogans*	-	-
**Rat 81**	*RN*	Male	Adult	4 urban	*L. interrogans*	-	-
**Rat 84**	*RN*	Male	Adult	4 urban	-	-	*L. interrogans*
**Rat 85**	*RN*	Male	Adult	4 urban	*L. interrogans*	-	*L. interrogans*
**Rat 91**	*RN*	Male	Adult	4 urban	*L. interrogans*	-	*L. interrogans*
**Rat 92**	*RN*	Female	Adult	4 urban	*L. interrogans*	-	*L. interrogans*
**Rat 93**	*RN*	Male	Adult	3 urban	*L. interrogans*	-	-
**Rat 95**	*RN*	Female	Adult	3 urban	*L. interrogans*	-	-
**Rat 96**	*RN*	Female	Adult	3 urban	*L. interrogans*	-	-
**Rat 97**	*RN*	Male	Adult	3 urban	-	-	*L. interrogans*

[a] *RN : Rattus Norvegicus, RR :Rattus Rattus*

[b] Site1: 36°27'45''N2°50'15''E, Site 2: 36°29'57''N2°50'43''E, Site 3: 36°28'02.6''N2°49'16.3''E, Site 4: 36°28'52.8''N2°51'04.5''E, Site 5: 36°31'39.4''N2°53'26.4''E, Site 6: 36°29'12''N2°48'21''E, Site 7 : 36°30'28''N2°53'48''E, Site 8: 36°29'51''N2°45'29''E, Site 9 : 36°27'24''N2°48'47''E.
(*)*Leptospira* species identified by sequencing after conventional PCR confirmation.
