# Supplementary figures and images for: First Molecular Detection of Pathogens Leptospira in Common Rodent Captured in North Algeria Urban Areas
Source: Trop Med Infect Dis. 2022 Oct 29;7(11):335. doi: 10.3390/tropicalmed7110335 (PMC9693108; doi:10.3390/tropicalmed7110335)

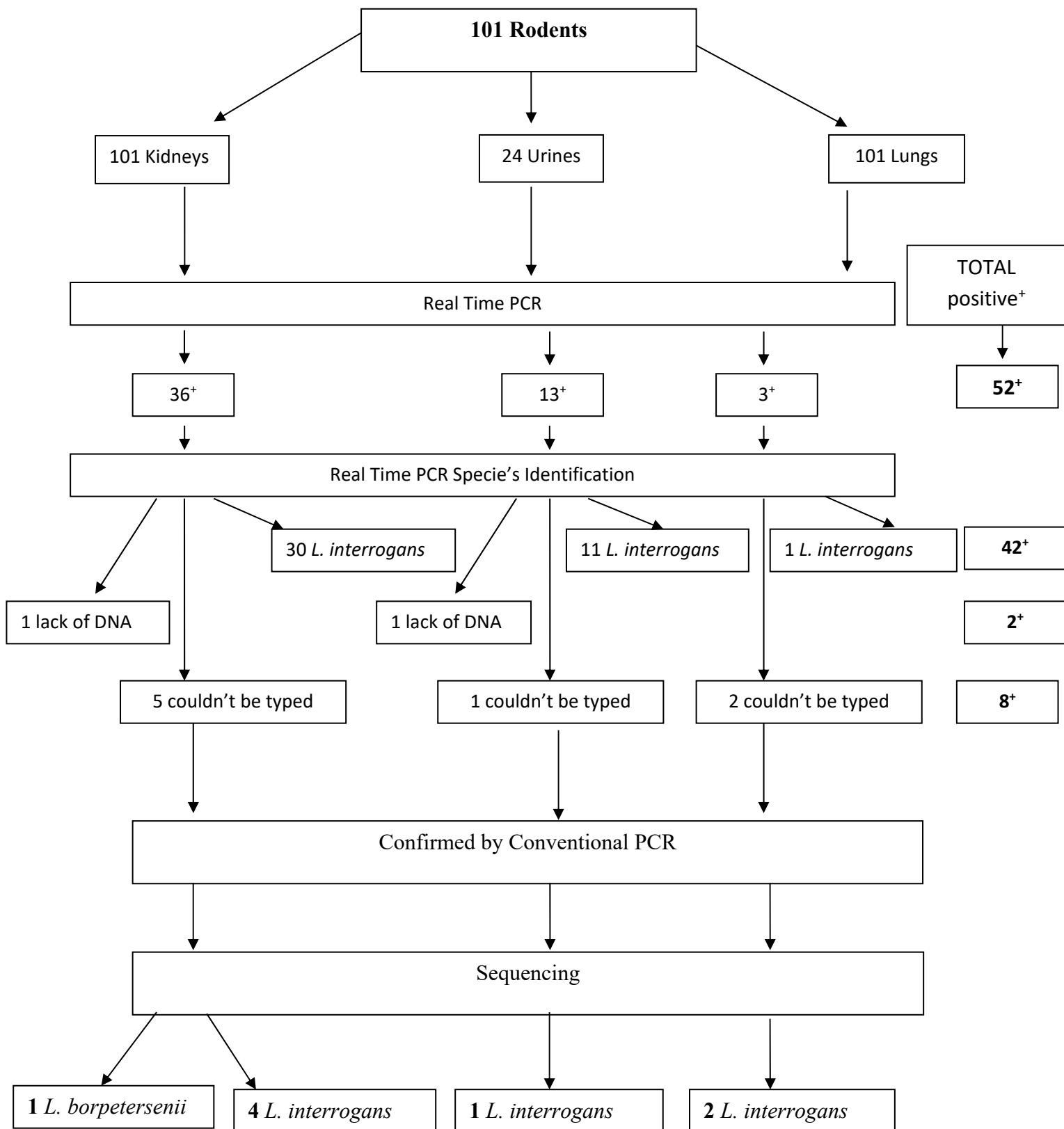

**Figure S1. Flow diagram summarizing all PCRs reactions.**

Supplement: Supplementary file 1 [file tropicalmed-07-00335-s001.zip › Figure S1.pdf]
